# Supplementary material for: Do parents counter-balance the carbon emissions of their children?
Source: PLoS One. 2020 Apr 15;15(4):e0231105. doi: 10.1371/journal.pone.0231105 (PMC7159189; doi:10.1371/journal.pone.0231105)
Supplement: S1 Appendix — (DOCX) [file pone.0231105.s001.docx]

**S1 Appendix A - Data.**

We begin by describe how we calculated household CO_2_ emissions. We first estimated household quantities consumed of goods and services and then matched consumption data with corresponding CO_2_ emissions. We used household expenditure data from Statistics Sweden for 2008-2009. Statistics Sweden samples 4,000 households in which at least one person was of age 0-79 years old. Statistics Sweden draws a random sample of households over the 52 starting weeks of household participation. Participates keep a diary for 14 days (or, alternatively, report receipts directly to Statistics Sweden) and are interviewed via phone. To collect data on household expenditures on goods and services purchased on a more infrequent basis (housing, holiday homes, furniture, cars, insurance and travel), Statistics Sweden conducts interviews and asks households about their expenditures during the last 12 months.

Statistics Sweden has matched participating households with register data on disposable income (including both labor income and government transfers).

Our data contains detailed expenditure information on goods and services that constitute the vast majority of household CO_2_ emissions, classified according to the international COICOP classification system on a four-digit level, see Table 3 and 4. It includes expenditures of food groups (including alcoholic and non-alcoholic beverages), transportation, clothing (including shoes) and housing.

We calculated quantities consumed by dividing expenditures by 2009 prices for all expenditure items. We then matched each item with their CO_2_ emissions per unit. We used several different data sources for this matching. Below, we describe in detail the data used for each broader expenditure category (food, transportation, clothing and housing), starting with the larger expenditure categories.

*Food and non-alcoholic beverages*. To calculate quantities consumed of food items consumed at home, we use detailed price data entailed in Consumer Price Index, collected by Statistics Sweden. For food consumed at home, we use CO_2_ emissions reported by Röös et al. (2014). These calculations are based on life cycle analysis (LCA). For food consumed away from home, households in the expenditure survey do not specify individual items purchased. For this group of food consumption, we use the CO_2_ emissions calculated by Statistics Sweden in their environmental accounts, which are based on expenditure shares of food away from home (Carlsson, Palm and Wadeskog, 2006). In other words, it is assumed that all households consume the average Swedish restaurant meal, and that emissions from food away from home vary cross households due to their variation in expenditures on food away from home.

*Clothing and footwear*. For this expenditure category, we use CO_2_ emission data from Statistics Sweden. Households do not specify individual items purchased of clothes and shoes. For this expenditure group, as with food away from home, we therefore use Statistics Sweden’s (Carlsson, Palm and Wadeskog, 2006) specification of CO_2_ emissions based on expenditure shares.

*Heating and electricity*. The electricity price is based on the average 2009 electricity spot price plus taxes. We use CO_2_ emissions from electricity as calculated by Swedenenergy (1 kWh emits 20g CO_2_). We use the figure for a normal year with electricity production based on domestic production (hydropower and nuclear power). The same number is also reported by Kellberg, C. (2016). Energy supply for heating of apartment buildings mainly consist of district heating. Energy usage for heating for households living in apartments (rental or own) is calculated as a fixed percentage (15 percent) of their rental cost in our main analysis, following Silverfur and Sjöberg (2015). Prices for district heating, both for apartments and single-family homes, are based on data from a market survey by ‘Energiforetagen’. See link https://www.energiforetagen.se/statistik/fjarrvarmestatistik/fjarrvarmepriser. The link entails prices for 2014 and 2015. Prices used in our analysis are revised for 2009, using price indices for district heating. Prices are higher for district heating in single family homes, compared to prices of district heating in apartments, due to price variations over the size of district heating customers.

Energy supply for single-family homes include sources other than district heating. Prices for pellets in 2009 varied between (SEK0.50-0.70/kwh), as reported by Lapplands Kommunalforbund (Energi- och Klimatradgivning). On Dec 31 2009, USD 1 = SEK 7.65, and EUR 1 = SEK 10.63. We use the average price (i.e., SEK0.60/kwh). CO_2_ emissions from pellets are from The Swedish Association of Public Housing Companies (SABO), also stated per Kwh. Both the oil price and CO_2_ emissions from oil is from the Swedish Petroleum and Bio Fuel Institute (Svenska Petroleum & Biodrivmedel Institutet [http://spbi.se/blog/faktadatabas/artiklar/berakningsmodeller/]). Although commonly used in many other countries, natural gas is a highly uncommon energy source in Sweden and therefore not part of our data.

*Transportation*. Most of transportation pertains to car trips, meaning CO_2_ emissions mainly pertain to car fuel (primarily gasoline, but also diesel and other fuels, like ethanol). In our sample, 86.1 percent of households report expenditures on car fuel. To calculate CO_2_ emissions from car transports, we again use prices and CO_2_ emissions from the Swedish Petroleum and Bio Fuel Institute (Svenska Petroleum & Biodrivmedel Institutet), see previous footnote. The average gasoline price in 2009 was SEK12.06/liter. For bus and taxi we use CO_2_ emissions from The Swedish Public Transport Association. CO_2_ emissions for buses differ depending on if the bus is part of the public transport system and if the bus is a (often long-distance) travel mode (where taxi also is included in the expenditure survey). Prices for bus trips are based on price in SEK/km, for a variety of short and long distance trips. The lower price interval bound is almost the same as the gasoline price/litre, which we choose to use in the main analysis, while performing robustness checks where the bus transport price is increased. CO_2_ emissions from domestic train trips is estimated to 1.1 grams of CO_2_/passenger km, which is calculated as the electricity usage 0.055 kWh/passenger km times the CO_2_ emissions from electricity usage 20 grams/kWh (0.055*20 = 1.1). The information about electricity usage is given by Sweden’s largest train operator SJ (<https://www.sj.se/en/about/about-sj/climate-friendly.html>) and corresponds to the electricity usage for an X2000 train at average occupancy. To get a measure of expenditures on train transportation, we divide CO_2_/passenger km from train transportation by the price travelers pay per km (SEK 1.30 per km) (Nelldal et al. 2013). CO_2_ emissions in gram per SEK spent on train transport then results in 0.84 g/SEK.

We do not use prices for flights or boat trips, but calculate CO_2_ emissions directly by assuming that the flight trip is a domestic flight in Sweden, between the two major cities (Gothenburg and Stockholm) www.klimatbalans.se/neutralisera/resor.html. CO_2_ emissions for boat trips are based on CO_2_ emissions from a cruise Stockholm-Helsinki [http://www.utslappsratt.se/berakna-utslapp/berakning-av-utslapp-fran-batar-och-fartyg/]. Only 2.1 percent of our sample reports expenditures for boat trips, and only 0.9 percent of the sample households report expenditures on flight transportation. These low numbers likely result from the method by which data is collected – to report expenditures on boat or flight trips, those trips must have been undertaken by the household during the two weeks of dairy reporting. Expenditures on package trips (COICOP 096) are collected by interviews, and refers to expenses over the past 12 months. Twelve percent of the sample report expenditures on domestic package tours and 52.6 percent report expenditures on international package tours. According to the Swedish national travel survey RES 2005-2006 (SIKA 2007), the Swede’s made 13,5 million international trips in 2006. Of these, 24 percent were shorter trips (less than 100 kilometers) to neighboring countries. Denmark, Finland, Norway, Germany and Spain were the most visited countries. The nine most popular destinations were all European countries, USA was the tenth most popular destination. Three fourth of the trips lasted for more than one day. Trips with a duration more than one day, hade on average a duration of seven days. The most common type of accommodation were hotels. Based on aggregate data on number of trips and mode of transport to the ten most visited countries, own calculations show that the average transport emissions per trip amounts to 153 kg CO_2_.

Although flights emit substantial CO_2_ per kilometer, road transportations constitute the absolute majority of transports, such that flight CO_2_ emissions are only a minor share of total emissions from transportation. The Swedish Environment Protection Agency estimates that greenhouse gases from all flight transports (i.e., private, public and commercial) constituted 2.8 percent of total greenhouse gas emissions in 2015, compared to 93.8 from road transports ( www.naturvardsverket.se/Sa-mar-miljon/Statistik-A-O/Vaxthusgaser-utslapp-fran-inrikes-transporter/).
