# Supplementary material for: Do parents counter-balance the carbon emissions of their children?
Source: PLoS One. 2020 Apr 15;15(4):e0231105. doi: 10.1371/journal.pone.0231105 (PMC7159189; doi:10.1371/journal.pone.0231105)
Supplement: S2 Appendix — (DOCX) [file pone.0231105.s002.docx]

**Appendix B – Robustness checks**

We examine whether excluding all subjects of age 65 and older impacts our results. We find that some household members work past their retirement age, increasing the risk of including households in our sample who currently are childless, but previously had children. Now our total sample size drops to 2,614 from 2,692. We see our main result remains robust. For instance, if we use this reduced sample to replicate the regression in Table 5, we find that two adults in a household with children annually emit 3207.94 kg CO_2_, while a childless household with two adults annually emits 3207.94 -667.43 = 2,540.502. The coefficient for the childless two-adult household is both large (-667.43) and statistically significant (*P*-value = 0.003), and implies that, when excluding subjects 65 years and older, becoming parents increases a two-adult household’s annual emissions of CO_2_ by 26.27 percent (i.e., somewhat higher than the 25 percent reported in our main analysis). To further check the robustness of our result with respect age, we estimate models where the oldest individual in the household has an age below 55 and 45 year. The main result also remains robust for these regressions. When the oldest individual in the household has an age blow 55 years the coefficient for the childless two-adult household is -663.09 (*P*-value = 0.011), and when the age is below 44 years the coefficient is -607.67 (*P*-value = 0.050).

The Swedish population in rural (northern) areas is typically older, which could mean parents and non-parents live in different regions. This could affect our results, given prices differ across regions. We therefore examine if our results remain robust when excluding small towns and counties in northern Sweden from our analysis. Specifically, we estimated models where we removed (a) households living in small towns in the four most northern counties in Sweden (rural areas) and (b) households living in the four most northern counties in Sweden. We find that our results are robust also to these changes. For (a) the point estimate for “Two adults without children” is -675.75; *P*-value = 0.006, and for (b) it is -712.37; *P*-value = 0.005.

In addition, we have estimated models with regional dummy variables representing the seven regions East, Southeast, South, West, Middle, Smaller towns/rural areas northern Sweden, Larger towns/cities northern Sweden, per the regional divide by Statistics Sweden. We have also added dummy variables for each child in each age group. Including those dummy variables very marginally affects the point estimate for the variable “Two adults without children.” Further, the model specification with dummy variables for each child in different age groups generates a large number of insignificant parameter estimates for these variables.

For CO_2_ emissions from boat trips, we estimate our model based on the assumption that expenditures on boat trips consists of fuel (gasoline) for privately owned boats by the households, instead of assuming expenditures on boat trips consists of a cruise Stockholm-Helsinki. This marginally impacts our overall results – we then find that parenthood increases CO_2_ emissions by 25 percent, i.e., a somewhat smaller percentage change than the 25.75 percentage change reported in our main analysis. Although marginal, the only noteworthy on impact our results is that the difference in CO_2_ emissions from transportation between parents and non-parents in two-adult households becomes somewhat less significant. The estimated coefficient for the difference in CO_2_ emissions between two adults without children increases some in magnitude and its associated *P*-value is 0.049 (the same as in our main analysis, see Table 8). We in turn examined how sensitive the results were to assumptions made for energy usage from district heating, CO_2_ emissions from gasoline and the price of public transportation.

For energy usage from district heating, we explored the sensitivity of our results to our assumption about the district heating usage of households living in apartments. In our main analysis, we assume household energy usage from district heating is a fixed percentage of 15 percent of their rental cost. If we substantially increase this percentage share to 20 percent, we find our results are almost unchanged. With this revision to our assumption, the difference in overall CO_2_ emissions between parents and non-parents increases by a modest 3 kg/year, while the difference in CO_2_ emissions from heating and electricity remains non-significant, as reported below.

For CO_2_ emissions from gasoline, we estimate our model based on the assumption that gasoline emits 2.6 kg of CO_2_ per liter (the CO_2_ emission per liter of diesel fuel), instead of the assumption in our main analysis of 2.24 kg of CO_2_ per liter. This revision to our data has a margin impact on our results. The absolute difference in CO2 emissions between adults in a two-adult household with and without children increases to 781.2 annual CO_2_ emissions. Note, however, the observed absolute value of emissions from the parents also increases, resulting in an overall increase in CO_2_ emissions from parenthood of 27 percent. This does not contradict our main finding that adults with children have a larger footprint. Emissions from the sub-group transportation itself are similarly marginally affected. For the price of public transportation, we estimate our models with prices of public transportation that are 5 and 10 times higher than those in our baseline model. This does not impact our main results at all, even when the price is multiplied by a factor as large as 10. It also has a marginal effect on the results for the sub-group transportation.

For expenditures on package trips we estimate the same econometric model as for CO_2_ emissions, see Table 11. Package trips include a number of services (e.g., travel and accommodation), and the expenditure survey lacks information on the specifics of the expenditures on package trips (i.e., if it mainly pertains to travel, accommodation, or something else). We use expenditures as the dependent variable in the regression model and find that for expenditures on domestic package trips are no significantly different for two-adult households with and without children. The estimated difference in expenditure is SEK 16 (approximately 2 USD, with the 2009 USD/SEK exchange rate). However, for international package trips the regression results suggest that two-adult households without children spend SEK 3,357 *more* than two-adult households with children, i.e., non-parents make more international trips. Calculations in Appendix A suggest that an international trip emits about 153 kg CO_2_ due to transportation. Surveys on Swedes’ expenditures on domestic tourism (SAEG, 2016) show that about one third of the expenditures are on transportation. If we assume the same proportion for international trips, we have that one third of the SEK 3,357 is spent on gasoline (the price on gasoline was SEK 12.06 per liter in 2009), which yields that a two-adult households without children emits 207.6 kg more CO_2_ than two-adult households with children on international package trips. If we would instead assume that half of the SEK 3,357 is spent on gasoline, CO_2_ emissions from international package trips would be 311.7 kg higher for non-parents. This number is still lower than the difference in CO_2_ emissions between two-adult households (396.32 kg) that we estimate for transportation in Table 8. If we include both domestic and international trips in our analysis, the difference in CO_2_ emissions from transportation between two-adult households with and without children is reduced to 188.7 kg, but our overall result that CO_2_ emissions from households with children are larger than those from non-parents remains.

**References cited only in the appendices**

Kellberg, C. (2016). Klimatpåverkan och växthusgaser? Svensk Energi. www.svenskenergi.se/Elfakta/Miljo-och-klimat/Klimatpaverkan/ (2017-01-10)

Nelldal, B-L., Troche, G. & Fröidh, O. (2013). Development of supply and prices for railway lines in Sweden 1990-2013 and deregulation and competition and development of regional traffic. Royal Institute of Technology (KTH). ISBN 978-91-87353-31-4

Röös, E., Sundberg, C., & Hansson, P. A. (2014). Carbon footprint of food products. In *Assessment of Carbon Footprint in Different Industrial Sectors, Volume 1* (pp. 85-112). Springer Singapore.

SIKA. (2007). RES 2005-2006 Den nationella resvaneundersökningen. SIKA Statistik 2007:19.

Silverfur, R. and Sjöberg, A. (2015) Fjarrvarme och miljo, Fastighetsagarna Sverige, Article number 803080, http://www.fastighetsagarna.se/webbshop/rapporter/miljo-och-energi/fjarrvarme-och-miljo-2015.
