# Supplementary material for: Do parents counter-balance the carbon emissions of their children?
Source: PLoS One. 2020 Apr 15;15(4):e0231105. doi: 10.1371/journal.pone.0231105 (PMC7159189; doi:10.1371/journal.pone.0231105)
Supplement: Attachment — Submitted filename: Review report on PONE-D-19-31142.pdf [file pone.0231105.s003.pdf]

Review report on:

"Do parents counter-balance the carbon emissions of their children?"

The question posed in this paper is whether becoming a parent makes an adult "greener" (in terms of CO_2 emissions). This question is posed in a Swedish context.

To answer this question a detailed microdata data on Swedish household consumption expenditure is combined or matched with CO_2 emission data. Since the microdata set includes various household characteristics, including number of adults and children in the household, they are able to calculate the CO_2 "footprint" for each type of household, and therefore able to test the effect of parenthood on household CO_2 emissions.

More precisely, as is stated on line 57-59, the question is whether parents themselves change their consumption choice and hence CO_2 emissions due their reproduction choice.

The results from the analysis shows that parenthood seems to affect emissions in the sense that childless households emit less CO_2 than households with children even if number if children is controlled for. This would imply that parenthood increases average adult CO_2 emissions. Therefore, it is claimed that having children increases CO_2 both by adding more people to the household, and by increasing emissions from the people in the household. Another way to put it, I guess, is that the composition of the consumption bundle is different, depending on whether there are children in the household or not (income is controlled for).

The paper is well written, and the statistical analysis seems appropriate given the data at hand. My main questions and comments concerns the research question itself, and the data that is used in the analysis.

General comments

There is, in my opinion, no good and clear motivation why the particular question is of interest. Given knowledge of how reproduction choice affects emissions, what do we do with such knowledge? I think that this should be made very clear in the paper. What the results show, as I understand, is that preferences differs between households, depending on whether they have children or not, implying that they allocate their budget differently. But, so what?

I guess we can do a similar descriptive analysis with respect to house-owner versus non-house-owner, or "pet" vs "non-pet" owners. But also in those cases we must motivate why this is important knowledge.

In the paper it is mentioned that it is two countervailing forces that may be at work when it comes to differences in emissions between parents and non-parents. One is that becoming parents may lead to focus more on the future, wanting their kids to have a good life and therefore try to contribute to less climate change. The other force that may go in the other direction is that parenthood may necessitate a more carbon intensive consumption bundle, especially when it comes to food and transport consumption. However, this study does not separate these two effects, but estimates a net effect only. Therefore, I cannot see how something can be said whether "parents

themselves become greener" (line 93). If it had been possible to separate these two forces it would certainly been interesting.

Still I can see a potential motivation, which is not mentioned. From the results it seems clear that households with kids have a more carbon intensive consumption bundle. It means, I guess, that the current carbon tax in Sweden tend to be carried by households with kids to a larger extent. This however is not discussed at all in the paper.

In summary I have some problems to see the motivation behind the analysis in the paper. In my opinion this has to be clarified. Related to this, given the results that parenthood implies a different budget allocation than non-parenthood; is that a problem? what to do with such information?

Specific comments

Section 2, data:

- Line 123 states that the data contain "household quantities consumed of goods and services". Is this really the case? As I understand from appendix A the consumption data is in expenditures (Swedish crowns), and that quantities are calculated by dividing expenditure with the relevant price. As I understand the prices used are the same for all households in the sample. An assumption that is made, then, is that all households face the same price for all the relevant goods and services. Is this really a reasonable assumption? Since emissions are directly connected to quantities by an input-output coefficient having the correct price becomes very crucial, I guess.
- Sweden is a rather large (at least long) country, and I would suspect that there is a non-trivial price variation for several of the relevant goods that may give biased emission estimates on the household level. Food prices, for example, varies I guess between rural areas and bigger cities due to differences in access to big low-price supermarkets. Electricity and fuel prices are other examples.
- The electricity price used in the paper is the spot price (day-ahead price) plus taxes. This however is not the price faced by the household since they buy electricity from retailers who offers many different types of contracts with varying prices. In addition, and more important, is that the distribution tariff has to be added and it varies substantially depending on household location and type of housing. Furthermore, the electricity tax also differs depending on household location. Households in the north of Sweden has a substantially lower consumption tax than households in the south. Taken together this means that there may be substantial price variation between different households also for electricity.
- The price used for bus trips is also, as I understand, the same for all households. Considering household location, I would guess that the type of bus trip differs quite a lot, and hence the price per km. Taken together these potential problems may lead to biased emission estimates. At least this issue should be discussed.

- I have a similar problem with trips abroad. There I'm not sure I understand how emissions are calculated, and if and how household composition affects emissions.
- Emissions from food consumed away from home is another problem, I can imagine. On lines 521-525 in appendix A it is stated that there is no data on the specific items consumed. Does this means that a dollar spent on food away from home results in the same amount of emissions, irrespective of type of household? If so, is this a potential problem?
- Emissions from electricity use in the household is assumed to generate 20 gram CO2 per KWh, while a KWh used by trains generates zero emissions. I simply do not understand this, makes no sense to me. It should also be explained where the 20 gram CO2 come from, considering the footnote 7 in appendix A.
- Descriptive statistics are displayed in Table 1 on page 7. Here it becomes unclear to me if the analysis is based on total CO2 emissions from household consumption (and hence total consumption expenditure), or only a subset consisting of food, transportation, electricity and heating? My interpretation of footnote a in Table 1 is that it is only the subset that is considered. On the other hand, line 161 on page 9 indicate that also clothing and shoes are included. I think all this has to be clarified. What is included, and if it is not total consumption that is included, why so and how large share of total consumption expenditure and emissions does the subset stand for?
- A final question concerning data concerns the way data is collected. As I understand (line 564-565) data is collected through a 2 week diary for each households. This may obviously create some problems concerning goods that households buy less frequent, such as flight and boat trips as mentioned. I guess this problem may arise also for other goods. Is this corrected for in the data somehow by Statistics Sweden, or is it handled somehow in the estimation process?

Section 2, Behavioral model:

- To call the econometric model "behavioral model" is in my opinion somewhat misleading. Household behavior is for me related to consumption choices, which is not explicitly considered here. I would just call it "econometric specification".
- Related to this, why isn't a "behavioral model" in the form of a demand system specified and estimated? The data for doing this seems to exist. In my opinion this would lead to more insight into household choice and drivers for emissions.
- Disposable income is used as independent variable. An alternative would be to use total expenditure. I do not see any discussion about this. Using disposable income implies, as I understand, an implicit assumption that consumption of non-durable goods are inseparable from consumption of durables and savings. It is however not unreasonable to believe that consumption of the non-durables considered here is separable from consumption of durables and savings, which would then motivate using total expenditure on the non-durables considered.
- Income squared is added as an independent variable, which seems reasonable. The basic motivation is that it allows for non-linear Engle curves, but also that it

provides an empirical test of the Environmental Kuznets curve based on microdata, which according to the paper is rare. However, I found at least one paper on this, that seems to be based on essentially the same data (Brannlund & Ghalwash, 2008).[1]

- What also would have been reasonable is to allow the number of children have a non-linear effect on emissions (and expenditure), due to scale effects with respect to number of children (see for example Deaton and Paxton, 1998[2]).
- There is no control variable for household location. From the literature we know that there are quite big differences in consumption patterns for rural and city households. Is the reason lack of data?

[1] Brännlund, R. and Ghalwash, T. (2008). The income–pollution relationship and the role of income distribution: An analysis of Swedish household data. Resource and Energy Economics, 30, 369-387.

[2] Deaton, A. and Paxson, C. (1998). Economies of scale, Household Size, and the Demand for Food. Journal of political economy, 106, 897-930.
