# Supplementary material for: Do parents counter-balance the carbon emissions of their children?
Source: PLoS One. 2020 Apr 15;15(4):e0231105. doi: 10.1371/journal.pone.0231105 (PMC7159189; doi:10.1371/journal.pone.0231105)
Supplement: Attachment — Submitted filename: Response to Reviewers.docx [file pone.0231105.s004.docx]

*Dear Dr Aguilar,*

*We would like to thank you and Reviewer 2 for excellent comments on our manuscript. The paper has greatly benefited from addressing your and Reviewer 2’s thoughtful and detailed comments. In particular, we have strengthened the motivation for the paper, are more careful with statements that we measure the effect of “becoming” a parent (as opposed to “being”) a parent, and have substantially edited and extended discussions on data and the methods section, as well as conducted numerous additional robustness checks.*

*In addition to addressing the specific comments from yourself and Reviewer 2, we have:*

- *Corrected the occasional typo and grammatical error in the manuscript.*
- *Added information on how to access the data is included in the data availability statement within the submission system.*

The household expenditure data (from the Household budget survey 2008 and 2009) that is used for this article is provided by Statistics Sweden. In Sweden, data for individual respondents (microdata) are protected by the Secrecy Act. Researchers can apply for access to microdata for use in specified research projects. The system for researchers’ access to microdata stored at Statistics Sweden is called Microdata Online Access (MONA). Users who receive deliveries via MONA can process the data over an internet connection without microdata ever leaving Statistics Sweden. Contact information to apply for access to (MONA) microdata: e-mail mikrodata@scb.se, phone +46 10 479 50 00, website: www.scb.se/en/services/guidance-for-researchers-and-universities/

*Below, we address each comment in turn.*

**Editor comments:**

**Two reviewers have offered their assessment to this submission. While one suggested its acceptance as it is, the second reviewer offered a detailed and constructive criticism of this empirical application. Among the reviewer's observations I highlight the motivation for this study, additional comments regarding the limitation of the data and econometric model, and the consideration of additional explanatory variables within their OLS regression.**

**All of Reviewer 2's comments are constructive, hence, addressing them will result in a much improved version. For instance, it seems that within the current model the authors could also easily control for rural/urban effects. As pointed by Reviewer 2 as well it seems the current model does not quiet match the profile of a behavioral one. For instance, one would expect attitudes, social norms, among other constructs to help explain the actual behavior in this case.  The authors should reconsider labeling their model this way.  Some additional details on the model estimation should be provided such as the test-statistic used to justify the use of robust standard errors. The authors should also offer a test for normality in the distribution of their dependent variable. Why the reporting of 'adjusted' R-squared if the model seemed to be estimated using OLS? A word on the limitations of the econometric model, inclusive of relatively low R-squared values, is warranted.**

*We thank the Editor for these very helpful comments. We address the comments that overlap with Reviewer 2 in our responses to Reviewer 2 below. The Editor individually also makes important and helpful observations about the reporting of our results. We address these here:*

**Test-statistic used to justify the use of robust standard errors.**

*To show the justification of the use of robust standard errors in our estimations, we have added results from a Lagrange multiplier test for heteroscedasticity to all regression results. These results are reported in each table reporting on regression results, i.e., Table 5 to Table 11.*

**The authors should also offer a test for normality in the distribution of their dependent variable.**

*We are grateful for this important suggestion. We have added the following to the text in section 2 Data and Econometric Specification (page 14):*

For estimation purposes we use a Least Squares estimator, i.e., an estimator that is unbiased, even if the residuals or the dependent variable are not normally distributed. We tested the dependent variable total CO2 emissions from consumption for normality using a Kolmogorov-Smirnov test, and find that it is not normally distributed (test statistic = 0.039; *P*-value<0.001).

**Why the reporting of 'adjusted' R-squared if the model seemed to be estimated using OLS?**

*The ‘adjusted’ part accounts for the number of explanatory variables included in the model. We choose to use the adjusted R-squared, instead of R-squared, since we aim to have a parsimonious parameter/model specification that captures the main explanatory variables. (For all of our regressions, the adjusted R-squared is very similar in magnitude to the R-squared value, and for the majority of our results, the adjusted R-squared measure is marginally lower than the R-squared. This is also shown by the regression results reported below in response to Reviewer 2’s comments.)*

**A word on the limitations of the econometric model, inclusive of relatively low R-squared values, is warranted.**

*We discuss limitations of the econometric model below. The main limitation is that we do not have longitudinal data. This also explains our relatively low R-squared values, since models estimated on cross-sectional data typically generate substantially lower R-squared values than do models based on time series, or panel, data.*

*We have added the following to section 3. Results (page 20):*

The adjusted R-squared values suggest that the regression models for total CO_2_ emissions and emissions from food have the highest explanatory power, with adjusted R-squared values of 0.33. The models for emissions from meat, transportation and gasoline have adjusted R-squared values of around 0.17, suggesting there is more heterogeneity in emissions from these goods.

**I will also ask the authors to possibly refrain from suggesting that the effects tested are the result of "becoming a parent".  Such choice of words infers a degree of change in emissions by the same individual (or household) before/after children in a more traditional differences-in-differences approach or in some kind of panel data setting - which is certainly not the case in the current dataset.**

*We have revised the manuscript throughout to refrain from suggesting the effects result from “becoming” a parent, and instead focus our language on “being” a parent. These edits are conducted in all parts of the paper. To further strengthen this point by clarifying the limitations of our analysis and data, we have also added the following revised wording to the Introduction (pages 4-5):*

Our analysis relies on cross-sectional observational data. While the benefits of our data are the level of detail and the wide range of consumption types included in the data set, the drawback is that we cannot control for any potential self-selection into parenthood that is based on green preferences, which undermines our ability to suggest any causal effect between parenthood and green behavior. In theory, such self-selection could exist, i.e., people who are less “green” might self-select into parenthood, which in turn might bias our results. However, empirical evidence suggests self-selection into parenthood in general (i.e., not only as a result of any potential environmental concerns) is uncommon, and self-selection based on reasons that affect CO_2_ emissions is even more unlikely.

Some of the childless households in our data will likely have children later in life, some are involuntarily childless (just like some households with children likely did not plan to have children), while some actively chose to remain childless. Any self-selection affecting our results only pertains to the latter group—those choosing never to have children. This is, however, a small group—of childless households age 20-40, only five percent state they do not want to have children [22]. The average young Swedish childless two adult-household plans to have children in the future. Further, over 50 percent of older childless households said they tried, but failed to have children. Of particular relevance to our analysis, not all willingly childless households (i.e., all of the five percent of 20-40 year old childless households) choose to abstain from having children due to reasons that might affect CO_2_ emissions, which is the kind of selection we would worry about for our study. In fact, the main reasons against having children have been found to be emotional stress from having a child, career interference, and not wanting the responsibility of bringing up a child, while environmental concern (e.g., concern for overpopulation), or variables that might correlate with CO_2_ emissions (e.g., travel preferences), have not been found to significantly affect the decision to be childless [23]. Still, a cautionary note is granted. Although unlikely, self-selection into parenthood in our data that is based on, or correlates with, green preferences cannot be ruled out, meaning that we can say with confidence that our analysis succeeds in comparing CO_2_ emissions between parents and non-parents, while there is some uncertainty surrounding its success at identifying the effect of CO_2_ emissions on *becoming* a parent.

*Added reference:*

*Langdridge, D., Sheeran, P., and Connolly, K. (2005) Understanding the reasons for parenthood. Journal of Reproductive and Infant Psychology, 23(2), 121-133.*

*We have added the following to section 4. Concluding Remarks (page 24)*:

While the benefits of our data are their level of detail and the broad range of consumption types covered, a drawback is that the cross-sectional nature of the data disables analysis of causality between parenthood and the observed higher carbon emissions. That being said, self-selection into parenthood based on preference greenness is unlikely: self-selection into parenthood itself is rare [22], and self-selection based on green preferences even more so, to the extent that previous studies fail to detect any effect of the decision to become a parent from factors either directly or indirectly affecting CO_2_ emissions [23]. It is therefore not unreasonable to imagine that our results imply a causal link -- parenthood *makes* people browner. The larger footprint from parenthood arises mainly from higher transportation emissions and browner food.

**Reviewer 1:**

**No comments (suggests accept as is).**

**Reviewer 2:**

**”Do parents counter-balance the carbon emissions of their children?”**

**The question posed in this paper is whether becoming a parent makes an adult “greener” (in terms of CO2 emissions). This question is posed in a Swedish context.**

**To answer this question a detailed microdata data on Swedish household consumption expenditure is combined or matched with CO2 emission data. Since the microdata set includes various household characteristics, including number of adults and children in the household, they are able to calculate the CO2 “footprint” for each type of household, and therefore able to test the effect of parenthood on household CO2 emissions.**

**More precisely, as is stated on line 57-59, the question is whether parents themselves change their consumption choice and hence CO2 emissions due their reproduction choice.**

**The results from the analysis shows that parenthood seems to affect emissions in the sense that childless households emit less CO2 than households with children even if number if children is controlled for. This would imply that parenthood increases average adult CO2 emissions. Therefore, it is claimed that having children increases CO2 both by adding more people to the household, and by increasing emissions from the people in the household. Another way to put it, I guess, is that the composition of the consumption bundle is different, depending on whether there are children in the household or not (income is controlled for).**

**The paper is well written, and the statistical analysis seems appropriate given the data at hand. My main questions and comments concerns the research question itself, and the data that is used in the analysis.**

**General comments**

**There is, in my opinion, no good and clear motivation why the particular question is of interest. Given knowledge of how reproduction choice affects emissions, what do we do with such knowledge? I think that this should be made very clear in the paper. What the results show, as I understand, is that preferences differs between households, depending on whether they have children or not, implying that they allocate their budget differently. But, so what?**

**I guess we can do a similar descriptive analysis with respect to house-owner versus non-house-owner, or “pet” vs “non-pet” owners. But also in those cases we must motivate why this is important knowledge.**

*We are grateful to the Reviewer for pointing out that the motivation of the study needs clarification. The purpose of the study is to contribute knowledge on what it means to CO_2_ emissions when children are added to the world. It is known that, amongst any consumer decision, having a child is by far the one that has the greatest impact on CO_2_ emissions (followed by travelling decisions and food choices), see Murtaugh and Schlax(2009); Wynes and Nicholas (2017). However, given that having the child is life-changing to the parent, this transformational experience might significantly counterbalance or exacerbate the CO_2_ emissions from having the child.*

*Other decisions might also affect CO_2_ emissions, such as the ones noted by the Reviewer (e.g., pet or house ownership). However, parenthood seems uniquely interesting. First, it is likely that having a child is one of (if not the) most life altering decisions – it is transformational, meaning it changes who you are at the core or “change[s] what it is like to be you,” and might change your “core preferences” (Paul, 2015, pp.156). Having a child might therefore be more likely than any other consumer decision to profoundly change a person’s preferences, including those that affect green behavior. Second, most people do become parents, such that any altered green behavior due to parenthood might have large effects on total CO_2_ emissions. Third, if any changes in green behavior occurs due to parenthood, these might be possible to address with appropriate policies – e.g., policies that target the unique situation of parents.*

*Reference (found both in the previous and current version of the manuscript):*

*References:*

*Murtaugh, P. A., and Schlax, M. G. (2009) Reproduction and the carbon legacies of individuals. Global Environmental Change, 19(1), 14-20.*

*Paul, L. A. (2015). What you can’t expect when you’re expecting. Res Philosophica, 92(2), 149-170.*

*Wynes, S., and Nicholas, K. A. (2017) The climate mitigation gap: education and government recommendations miss the most effective individual actions. Environmental Research Letters, 12(7).*

*We have added the following to the Introduction (page 3):*

Parenthood has the potential to significantly affect climate change. While other consumer decisions might also affect CO_2_ emissions, it might be of particular interest to study the effect on the climate from parenthood. First, as opposed to most other consumer decisions, having a child changes people -- it changes people’s “core preferences” [3], therefore potentially also people’s green preferences. Second, most people become parents, such that any altered green behavior due to parenthood has the potential to significantly affect total CO_2_ emissions. Third, it seems entirely feasible to design policies that could mitigate any increased CO_2_ emissions from parenthood, by targeting the unique situation of parents.

**In the paper it is mentioned that it is two countervailing forces that may be at work when it comes to differences in emissions between parents and non-parents. One is that becoming parents may lead to focus more on the future, wanting their kids to have a good life and therefore try to contribute to less climate change. The other force that may go in the other direction is that parenthood may necessitate a more carbon intensive consumption bundle, especially when it comes to food and transport consumption. However, this study does not separate these two effects, but estimates a net effect only. Therefore, I cannot see how something can be said whether “parents themselves become greener” (line 93). If it had been possible to separate these two forces it would certainly been interesting.**

*We agree with the Reviewer that it would be interesting to take our analysis even further, and examine not only the net effect of the two countervailing forces at play, i.e., to also be able to directly identify these forces (to what degree people’s preferences change when they become parents, and to what degree they experience tighter budget constraints). Although uniquely detailed in other regards, our data unfortunately do not enable us to do so -- we can only observe choices, not preferences or constraints that determine those choices. From a climate impact perspective, the net effect is the most important. From a policy perspective, identifying the counter veiling forces would be helpful. We have strengthened the discussion about these counter veiling forces, and included more of previous work (research and reports) that examines either of these (note, no previous research addresses both), in the Introduction. We have also suggest disentangling these effects as a direction for future research in the Conclusions.*

*Specifically, we now state in the Introduction (pages 3-4):*

Whether parents mitigate or accentuate the increased emissions of their children is *a priori* ambiguous. Two countervailing forces are at work. First, the transformational experience of having a child. On having children, Paul (2015), pp.156, clarifies: “a personally transformative experience radically changes what it is like to be you, perhaps by replacing your core preferences with very different ones” [3]. Particularly relevant to our context, parenthood makes people worry more about the future [4,5], and increases the strength of preferences for environmental goods [6], which may suggest using less carbon-intensive goods and services to reduce future risks of climate change [3, 7-12]. But the flip side is that time constraints now become more binding and convenience may become more important —parents may need to be in more places in one day. They also need to feed more people. Eating more pre-prepared, red meat carbon-intensive meals may add convenience and save time. Parents’ environmental behavior may also be motivated by other factors, such as health and income [6,13-15].

*Added references:*

*Sundblad, E. L., Biel, A., and Gärling, T. (2007) Cognitive and affective risk judgements related to climate change. Journal of Environmental Psychology, 27(2), 97-106.*

*McCright, A. M., and Dunlap, R. E. (2013) Bringing ideology in: the conservative white male effect on worry about environmental problems in the USA. Journal of Risk Research, 16(2), 211-226.*

*Dupont, D. P. (2004) Do children matter? An examination of gender differences in environmental valuation. Ecological Economics, 49(3), 273-286.*

*Added to section 4. Concluding Remarks (page 24):*

What causes parents to emit more of CO_2_? The higher CO_2_ emissions from parenthood could be the net outcome of two previously documented counter-veiling effects: parents care more for the future [4,5], while being more pressed to satisfy immediate needs under tighter time constraints [24]. In this study, we cannot directly observe these underlying consumption motivations. While our study suggests the difference in CO_2_ emissions between parents and non-parents is substantial, and therefore might warrant policy makers’ attention, we encourage future research to examine the strength of the underlying factors that might cause the consumption differences.

**Still I can see a potential motivation, which is not mentioned. From the results it seems clear that households with kids have a more carbon intensive consumption bundle. It means, I guess, that the current carbon tax in Sweden tend to be carried by households with kids to a larger extent. This however is not discussed at all in the paper.**

*We appreciate this suggestion from the Reviewer. Our data suggest that families with children use transportation to a greater extent, and hence bear a larger fraction of the CO_2_ tax on petrol and diesel for transportation, compared to those without children. There is no significant difference in CO_2_ emissions from heating and electricity across families with and without children. (There is no carbon tax on other consumption that also largely contributes to emissions, such as food.) This could imply that families with children bear more of the carbon tax. To determine who bears the carbon tax burden, a paper on that topic would also require some additional knowledge on distributions of carbon tax revenues. It would be a different topic than the one we aim to address in this paper, but nevertheless interesting. We have added the following to section 4.Concluding Remarks (page 25):*

Finally, our results might also imply that there could be distributional effects from the Swedish carbon tax across families with and families without children, when controlling for the number and age of people in the family. A follow-up study might determine the incidence of carbon taxes across households with and without children.

**In summary I have some problems to see the motivation behind the analysis in the paper. In my opinion this has to be clarified. Related to this, given the results that parenthood implies a different budget allocation than non-parenthood; is that a problem? what to do with such information?**

*We address the motivation of the study above. The Reviewer also wonders what we can do with the information generated by the study? We appreciate the Reviewer further pushing us towards more clearly justifying the study and explain how any knowledge gained from the study might be useful. This is further clarified in the following addition to section 4. Concluding Remarks (page 24-25):*

What causes parents to emit more of CO_2_? The higher CO_2_ emissions from parenthood could be the net outcome of two previously documented counter-veiling effects: parents care more for the future [4,5], while being more pressed to satisfy immediate needs under tighter time constraints [24]. In this study, we cannot directly observe these underlying consumption motivations. While our study suggests the difference in CO_2_ emissions between parents and non-parents is substantial, and therefore warrants policy makers’ attention, we encourage future research to examine the strength of the underlying factors that might cause the consumption differences. Such knowledge might be important in identifying policies that effectively reduce the carbon footprint from parents, e.g., policies that alleviate their time crunch -- increased access to child care, increased work hour flexibility for parents, and so on.

Further, the differences in food consumption between parents and non-parents might be helped by recent innovations in developing meat substitutes that “taste like meat,” and hence might appeal to everyone in the family [52]. Such meat substitutes are still relatively expensive -- government subsidies of meat substitutes could help speed up the transition from meat to vegetarian proteins, especially for families with children, given they might be particularly constrained by satisfying heterogonous food preferences within the family.

*Added references:*

*Keefe, L. M. (2018). # FakeMeat: How big a deal will animal meat analogs ultimately be?. Animal Frontiers, 8(3), 30-37.*

*McCright, A. M., and Dunlap, R. E. (2013) Bringing ideology in: the conservative white male effect on worry about environmental problems in the USA. Journal of Risk Research, 16(2), 211-226.*

*Sundblad, E. L., Biel, A., and Gärling, T. (2007) Cognitive and affective risk judgements related to climate change. Journal of Environmental Psychology, 27(2), 97-106.*

**Specific comments**

*Section 2, data:*

- **Line 123 states that the data contain “household quantities consumed of goods and services”. Is this really the case? As I understand from appendix A the consumption data is in expenditures (Swedish crowns), and that quantities are calculated by dividing expenditure with the relevant price. As I understand the prices used are the same for all households in the sample. An assumption that is made, then, is that all households face the same price for all the relevant goods and services. Is this really a reasonable assumption? Since emissions are directly connected to quantities by an input-output coefficient having the correct price becomes very crucial, I guess.**

*We thank the Reviewer for this insightful comment. The Reviewer’s observation is correct --we divide expenditures by prices to calculate quantities consumed. As noted by the Reviewer, this means that there might be deviations (positive or negative) between the true quantities consumed and the quantities calculated in this study. However, the main question is whether there are systematic differences in prices faced by parents and non-parents. There is no evidence of such systematic differences, which implies that the error that we have in our calculations for parents and non-parents should be random and our point estimates in the econometric models should be unbiased. To address this concern, we have added the following text at the end of section 2. Data and Econometric Specification (page 14):*

Note that since we assume prices are the same for all households, our model might over or under estimated CO_2_ emissions for a specific household. However, there is no systematic difference in prices across parents and non-parents. We therefore expect our econometric models presented below to generate an unbiased point estimate of the difference in CO_2_ emissions between parents and non-parents.

*Further, on line 123 in the previous version of the manuscript we have changed “household quantities consumed” to “household expenditures on” in the following sentence (page 7):*

We summarize our unique data below, and Appendix A provides the specific details. Our data contained household expenditures on goods and services from a set of 4,000 households surveyed by Statistics Sweden for 2008-2009.

- **Sweden is a rather large (at least long) country, and I would suspect that there is a non-trivial price variation for several of the relevant goods that may give biased emission estimates on the household level. Food prices, for example, varies I guess between rural areas and bigger cities due to differences in access to big low-price supermarkets. Electricity and fuel prices are other examples.**

*We thank the Reviewer for this thoughtful remark. It is true that there are regional variations in the prices. Although, there is no reason to expect price difference between parents and non-parents in the same region, regional price difference could be a problem for our analysis if households with children and households without children are living in different regions.*

*In Sweden, the population is older in the rural areas. Older households are more likely to be classified as non-parents, since their children have moved out from the household. In the robustness analysis we remove elderly people (people above 55 and 45 years of age), which makes the sample more homogeneous from a regional perspective. As the robustness analysis in the previous version of the manuscript shows, we get the same overall result when we remove elderly people from the sample. In addition, we control for age in the regression analysis.*

*To further address this issue, we have conducted additional robustness checks by estimating our model after having removed households living in:*

i) *small towns in the four most northern counties in Sweden (rural areas)*
ii) *the four most northern counties in Sweden.*

*We find that our results are robust also to these changes. For (i) the coefficient for “Two adults without children” is -675.75; P-value = 0.006, and for (ii) the “Two adults without children” is -712.37; P-value = 0.005.*

*We have added the following to Appendix B -- Robustness checks (page 38-39):*

The Swedish population in rural (northern) areas is typically older, which could mean parents and non-parents live in different regions. This could affect our results, given prices differ across regions. We therefore examine if our results remain robust when excluding small towns and counties in northern Sweden from our analysis. Specifically, we estimated models where we removed (a) households living in small towns in the four most northern counties in Sweden (rural areas) and (b) households living in the four most northern counties in Sweden. We find that our results are robust also to these changes. For (a) the point estimate for “Two adults without children” is -675.75; *P*-value = 0.006, and for (b) it is -712.37; *P*-value = 0.005.

- **The electricity price used in the paper is the spot price (day-ahead price) plus taxes. This however is not the price faced by the household since they buy electricity from retailers who offers many different types of contracts with varying prices. In addition, and more important, is that the distribution tariff has to be added and it varies substantially depending on household location and type of housing. Furthermore, the electricity tax also differs depending on household location. Households in the north of Sweden has a substantially lower consumption tax than households in the south. Taken together this means that there may be substantial price variation between different households also for electricity.**

*We are grateful for this insightful comment by the Reviewer, and the Reviewer makes an accurate description of the electricity price. Unfortunately, our data does not contain information about the type of contract the household has.*

*While CO_2_ emissions from electricity are imperfect from a regional perspective, key to our analysis is that there is no difference in prices across parents and non-parents. Still, to control for regional differences we have included regional dummy variables in our regression models as an additional robustness check and the results remain the same as in the model without regional dummy variables. The results from the regression models are shown below. In the regression models we control for the seven regions defined by Statistics Sweden (East, Southeast, South, West, Middle, Smaller towns/rural areas northern Sweden, Larger towns/cities northern Sweden). We use region Middle as the reference case in the below regressions.*

*We have added the following to Appendix B – Robustness checks (page 39):*

In addition, we have estimated models with regional dummy variables representing the seven regions East, Southeast, South, West, Middle, Smaller towns/rural areas northern Sweden, Larger towns/cities northern Sweden, per the regional divide by Statistics Sweden. We have also added dummy variables for each child in each age group. Including those dummy variables very marginally affects the point estimate for the variable “Two adults without children.” Further, the model specification with dummy variables for each child in different age groups generates a large number of insignificant parameter estimates for these variables.

- **The price used for bus trips is also, as I understand, the same for all households. Considering household location, I would guess that the type of bus trip differs quite a lot, and hence the price per km. Taken together these potential problems may lead to biased emission estimates. At least this issue should be discussed.**

*We thank the Reviewer for this observation. We carry out a robustness analysis in Appendix B to evaluate our assumption about the price for bus trips/public transport. We find that our results are robust with respect to changes in the ticket price. As noted above, we have also included regional dummy variables in our regression models. These models gives the same overall results as when we do not control for region.*

*We present the following text in Appendix B – Robustness checks (page 40)*:

For the price of public transportation, we estimate our models with prices of public transportation that are 5 and 10 times higher than those in our baseline model. This does not impact our main results at all, even when the price is multiplied by a factor as large as 10. It also has a marginal effect on the results for the sub-group transportation.

- **I have a similar problem with trips abroad. There I’m not sure I understand how emissions are calculated, and if and how household composition affects emissions.**

*For trips abroad (package trips) we carry out an analysis where we use expenditures as the dependent variable instead of CO_2_ emissions. The reason for this is that we lack information about the specific goods that are included in package trips expenditures. However, we know that for domestic tourism about one third of the expenditures are spent on transportation. In the robustness checks (Appendix B) we assume the same proportion for international package trips. (We also consider a case where we assume that 50 percent of expenditures is spent on transportation.)*

*The regression results suggest that a two-adult household without children spends SEK 3,357 more on international package trips than a two-adult household with children. If we assume that one third of the SEK 3,357 is spent on gasoline, a two-adult household without children would emit 207.6 kg more CO_2_ than a two-adult household with children on international package trips.*

*This number is then compared to the estimated CO_2_ emissions from domestic transportation in Table 8 to examine if the estimated emissions from trips abroad change our result (i.e., the result that emissions from transportation are larger for parents than non-parents). The difference in CO_2_ emissions between two-adult households with and without children in Table 8 is 396.32 kg, i.e., a two-adult household with children emits 396.32 kg CO_2_ more than a two-adult household without children on domestic transportation.*

*Although two-adult households without children emit more CO_2_ from trips abroad (207.6 kg), this number is still smaller than the difference in CO_2_ emissions (396.32 kg) from domestic transportation. If we include both domestic and international trips in our analysis, the difference in CO_2_ emissions from transportation between two-adult households with and without children is reduced to 188.7 kg, but our overall result that CO_2_ emissions from households with children are larger than those from non-parents remains.*

*We have added more detail to Appendix B – Robustness checks and also clarify this as follows in the main text, section 3. Results (page 22):*

For expenditures on package trips, Table 11 shows that a two-adult household without children spends SEK 3,356.52 more on international package trips annually compared to two adults in a household with children (*P*-value = 0.043). The expenditure data entails no information on the content of international package trips. However, travel surveys indicate that about one third of household expenditures on domestic trips are spent on transportation [51]. If the same proportion is spent on transportation for international package trips, this suggests that a two-adult household without children emits 207.6 kg more of CO_2_ compared to two-adults in a household with children, if making the assumption that all travel related expenditures are spent on gasoline. This implies our main result holds even if parents may emit less from international trips, compared to non-parents. Appendix B provides more information about the data and assumptions for Swedes’ international trips and CO_2_ emissions.

- **Emissions from food consumed away from home is another problem, I can imagine. On lines 521-525 in appendix A it is stated that there is no data on the specific items consumed. Does this means that a dollar spent on food away from home results in the same amount of emissions, irrespective of type of household? If so, is this a potential problem?**

*We thank the Reviewer for this thoughtful remark. Yes, that is the implication, since we use a common CO_2_ coefficient for expenditures on food consumed away from home. It means that the household buys the average meal that is produced in Swedish restaurants, and that the variation in emissions is a function of the household’s expenditures on food away from home. Since food consumed at home has a larger CO_2_ footprint for households with children, it might be that these households also have a larger footprint from meals consumed away from home. However, to the best of our knowledge, there is no study that has looked into that particular question. If households with children have a larger CO_2_ footprint per meal compared to households without children, we underestimate the CO_2_ emissions from households with children. This would give additional support for our results that parents are browner. However, since there is a lot of uncertainty related to this assumption we use a common CO_2_ coefficient for all types of households to keep the assumption(s) as neutral as possible. To clarify the above, we have added the following to Appendix A – Data (page 33):*

In other words, it is assumed that all households consume the average Swedish restaurant meal, and that emissions from food away from home vary cross households due to their variation in expenditures on food away from home.

- **Emissions from electricity use in the household is assumed to generate 20 gram CO2 per KWh, while a KWh used by train s generates zero emissions. I simply do not understand this, makes no sense to me. It should also be explained where the 20 gram CO2 come from, considering the footnote 7 in appendix A.**

*We thank the reviewer for notifying us that this needs further clarification. The reference for the 20 grams of CO_2_ emissions is given in footnote 2 in the Appendix A -- Data. We have also added a second reference to footnote 2. The new reference is:*

*Kellberg, C. (2016). Klimatpåverkan och växthusgaser? Svensk Energi.* [*www.svenskenergi.se/Elfakta/Miljo-och-klimat/Klimatpaverkan/*](http://www.svenskenergi.se/Elfakta/Miljo-och-klimat/Klimatpaverkan/) *(2017-01-10).*

*Footnote 7 has been deleted.*

*Further, we have changed the CO_2_ emission from train transports. In the new calculations we use the emission coefficient for electricity 20 grams of CO_2_ per KWh together with the electricity usage per passenger km. This results in 1.1 grams of CO_2_ per passenger km. To get a measure that is related to expenditures on train transportation we divide CO_2_/passenger km from train transportation by the price travelers pay per km. CO_2_ emissions in gram per SEK spent on train transport then becomes 0.84 g/SEK.*

*The following has been added to Appendix A – Data (page 35):*

CO_2_ emissions from domestic train trips is estimated to 1.1 grams of CO_2_/passenger km, which is calculated as the electricity usage 0.055 kWh/passenger km times the CO_2_ emissions from electricity usage 20 grams/kWh (0.055*20 = 1.1). The information about electricity usage is given by Sweden’s largest train operator SJ (<https://www.sj.se/en/about/about-sj/climate-friendly.html>) and corresponds to the electricity usage for an X2000 train at average occupancy. To get a measure of expenditures on train transportation, we divide CO_2_/passenger km from train transportation by the price travelers pay per km (SEK 1.30 per km) (Nelldal et al. 2013). CO_2_ emissions in gram per SEK spent on train transport then results in 0.84 g/SEK.

*Added reference to the Appendices:*

*Nelldal, B-L., Troche, G. & Fröidh, O. (2013). Development of supply and prices for railway lines in Sweden 1990-2013 and deregulation and competition and development of regional traffic. Royal Institute of Technology (KTH). ISBN 978-91-87353-31-4.*

- **Descriptive statistics are displayed in Table 1 on page 7. Here it becomes unclear to me if the analysis is based on total CO2 emissions from household consumption (and hence total consumption expenditure), or only a subset consisting of food, transportation, electricity and heating? My interpretation of footnote a in Table 1 is that it is only the subset that is considered. On the other hand, line 161 on page 9 indicate that also clothing and shoes are included. I think all this has to be clarified. What is included, and if it is not total consumption that is included, why so and how large share of total consumption expenditure and emissions does the subset stand for?**

*We thank the Reviewer for notifying us that the information in the note to Table 1 was incomplete. We have edited the note such that it now reads:*

^a^ Total CO_2_ emissions from consumption included in our analysis (food, transportation, electricity and heating, clothing and shoes).

*We have added the following to section 2. Data and Econometric Specification (page 10):*

These four expenditure groups account for about 85 percent of the households’ total CO_2_ emissions.

*With that addition, the text now reads:*

...we capture detailed data on four expenditure groups: (a) food and non-alcoholic beverages, (b) transportation, (c) clothing, including shoes, and (d) housing. Tables 3 and 4 summarize the expenditure groups and associated levels of CO_2_ emissions (Appendix A provides the specific matching numbers and sources). These four expenditure groups account for about 85 percent of the households’ total CO_2_ emissions.

- **A final question concerning data concerns the way data is collected. As I understand (line 564-565) data is collected through a 2 week diary for each households. This may obviously create some problems concerning goods that households buy less frequent, such as flight and boat trips as mentioned. I guess this problem may arise also for other goods. Is this corrected for in the data somehow by Statistics Sweden, or is it handled somehow in the estimation process?**

*To collect data on the households’ expenditures on housing, holiday home, furniture, car, insurance and travel, Statistic Sweden carries out interviews with the households and asks for their expenditures during the last 12 months. All expenditures in the survey (also those collected during the two week period) are then recalculated/aggregated to annual levels.*

*To address this, we have added the following text to Appendix A – Data (page 32):*

To collect data on household expenditures on goods and services purchased on a more infrequent basis (housing, holiday homes, furniture, cars, insurance and travel), Statistics Sweden conducts interviews and asks households about their expenditures during the last 12 months.

*Section 2, Behavioral model:*

- **To call the econometric model “behavioral model” is in my opinion somewhat misleading. Household behavior is for me related to consumption choices, which is not explicitly considered here. I would just call it “econometric specification”.**

*We are grateful to the Reviewer for this observation. We have followed the recommendation from the Reviewer to rename the model “econometric specification.” Both the heading of section 2 and the initial text for that section have been edited accordingly.*

- **Related to this, why isn’t a “behavioral model” in the form of a demand system specified and estimated? The data for doing this seems to exist. In my opinion this would lead to more insight into household choice and drivers for emissions.**

*We thank the Reviewer for the comment and suggestion. The model(s) that we estimate are closely related to demand models, but we do not include prices as explanatory variables in the econometric model, they are instead used to calculate the quantities that are consumed. We are interested in explaining CO_2_ emissions from different types of households and our econometric specification enables us to estimate that effect directly. An analysis that includes a demand model could be used to study, for instance, the impact of prices on CO_2_ emissions. Although analyses of that kind are interesting, they are outside the scope of this article.*

- **Disposable income is used as independent variable. An alternative would be to use total expenditure. I do not see any discussion about this. Using disposable income implies, as I understand, an implicit assumption that consumption of non-durable goods are inseparable from consumption of durables and savings. It is however not unreasonable to believe that consumption of the non-durables considered here is separable from consumption of durables and savings, which would then motivate using total expenditure on the non-durables considered.**

*We thank the Reviewer for this comment. By using disposable income, instead of total expenditures, we avoid unnecessary restrictions on the individuals’ preferences, such as assuming that the consumptions of non-durable goods are separable from durable goods. For instance, it is not evident that gasoline consumptions (a non-durable good) is separable from car ownership (a durable good). This type of discussion is common in studies were the model specification suggests total expenditures would be preferable to disposable income (which is not the case for our study), or the authors do not have access to disposable income, and therefore need to justify the use of total expenditures. As we aim to focus the discussion about the econometric model, we hope the Reviewer agrees with us that a discussion on this topic might be less needed.*

- **Income squared is added as an independent variable, which seems reasonable. The basic motivation is that it allows for non-linear Engle curves, but also that it provides an empirical test of the Environmental Kuznets curve based on microdata, which according to the paper is rare. However, I found at least one paper on this, that seems to be based on essentially the same data (Brannlund & Ghalwash, 2008).^1^**

*We thank the reviewer for notifying us of the reference Brännlund and Ghalwash (2008). We have added Brännlund and Ghalwash (2008) to the text of the manuscript, where we mention that our data support previous findings of the Kuznets curve (page 14). We have double-checked our general search for studies that estimate the Kuznets curve for Sweden (and other countries) based on micro-level data, and failed to identify any other studies.*

*Added reference*:

*Brännlund, R., and Ghalwash, T. (2008) The income–pollution relationship and the role of income distribution: An analysis of Swedish household data. Resource and Energy Economics, 30(3), 369-387.*

- **What also would have been reasonable is to allow the number of children have a non-linear effect on emissions (and expenditure), due to scale effects with respect to number of children (see for example Deaton and Paxton, 1998^2^).**

**^2^ Deaton, A. and Paxson, C. (1998). Economies of scale, Household Size, and the Demand for Food. Journal of political economy, 106, 897-930.**

*Please see comment below.*

- **There is no control variable for household location. From the literature we know that there are quite big differences in consumption patterns for rural and city households. Is the reason lack of data?**

*We thank the Reviewer for the two helpful comments above that pertain to the specification of the econometric model. To address these comments, we have re-estimated our models, see below. In these models we control for the region in which the households reside. We also include a non-linear specification for children in different age groups, represented by dummy variables for one child, two children and two or more children in different age groups. The results from these regression are presented below. As seen from the tables, the parameter estimate for the main variable of interest “Two adults without children” remains largely unchanged in all model specifications. The model with child dummies results in a fairly large number of insignificant point estimates. We have therefore chosen to keep our initial specification of the regression equation.*

*To communicate these insights, we have added the following to Appendix B – Robustness checks (page 39; repeated from above):*

In addition, we have estimated models with regional dummy variables representing the seven regions East, Southeast, South, West, Middle, Smaller towns/rural areas northern Sweden, Larger towns/cities northern Sweden, per the regional divide by Statistics Sweden. We have also added dummy variables for each child in each age group. Including those dummy variables very marginally affects the point estimate for the variable “Two adults without children.” Further, the model specification with dummy variables for each child in different age groups generates a large number of insignificant parameter estimates for these variables.

*In the regression results*

*Region_1 = East*

*Region_2 = Southeast*

*Region_3 = South*

*Region_4 = West*

*Region_5 = Middle (control region in the regression models)*

*Region_6 = Smaller towns/rural areas northern Sweden*

*Region_7 = Larger towns/cities northern Sweden*

**Table 5. Regression results, total CO_2_ emissions**

```
                             NONLINEAR LEAST SQUARES
                             =======================

 Parameter  				  Estimate      Error       t-statistic   P-value
 Constant   				 3513.80       323.440       10.8638       [.000]
 NUMBER OF CHILDREN 0-6 YEARS     187.508       115.147       1.62843       [.103]
 NUMBER OF CHILDREN 7-12 YEARS    422.494       112.796       3.74566       [.000]
 NUMBER OF CHILDREN 13-17 YEARS   502.575       123.797       4.05969       [.000]
 NUMBER OF CHILDREN 8-19 YEARS    203.507       218.404       .931791       [.351]
 TWO ADULTS WITHOUT CHILDREN      -717.983      236.598       -3.03461     [.002]
 ONE ADULT WITHOUT CHILDREN    	 -2330.72      254.866       -9.14488     [.000]
 ONE ADULT WITH CHILDREN   	-1885.64      207.908       -9.06956      [.000]
 AGE      				28.7789       5.34272       5.38657       [.000]
 Disp. Income				6.83946       .661856       10.3338       [.000]
 Disp. Income squared 		-.220133E-02  .259956E-03   -8.46809      [.000]

 Standard Errors computed from heteroscedastic-consistent matrix
 (Robust-White)

        Mean of dep. var. = 6891.78               R-squared = .328409
   Std. dev. of dep. var. = 3390.54      Adjusted R-squared = .325892
 Sum of squared residuals = .206831E+11        LM het. test = 41.5905 [.000]
    Variance of residuals = .774938E+07       Durbin-Watson = 1.87912 [<.002]
 Std. error of regression = 2783.77
```

**Table 5. Regression results, total CO_2_ emissions: control variables regional dummy variables**

```
                             NONLINEAR LEAST SQUARES
                             =======================

 Parameter  				  Estimate        Error       t-statistic   P-value
 CONSTANT   				   3784.15       343.052       11.0308       [.000]
 NUMBER OF CHILDREN 0-6 YEARS       185.036       114.684       1.61344       [.107]
 NUMBER OF CHILDREN 7-12 YEARS      388.843       112.364       3.46056       [.001]
 NUMBER OF CHILDREN 13-17 YEARS     490.876       123.185       3.98486       [.000]
 NUMBER OF CHILDREN 8-19 YEARS      124.528       217.429       .572731       [.567]
 TWO ADULTS WITHOUT CHILDREN     	   -723.415      234.697       -3.08234      [.002]
 ONE ADULT WITHOUT CHILDREN     	   -2274.85      252.175       -9.02093      [.000]
 ONE ADULT WITH CHILDREN   	   -1834.52      207.248       -8.85182      [.000]
 AGE      			          27.6816       5.32551       5.19793       [.000]
 DISP. INCOME         		   7.26953       .671592       10.8243       [.000]
 DISP. INCOME SQUARED         	   -.233668E-02  .272982E-03   -8.55983      [.000]
 REGION 1         			   -818.015      171.923       -4.75805      [.000]
 REGION 2         			   -397.534      178.581       -2.22607      [.026]
 REGION 3         			   -64.2113      224.982       -.285407      [.775]
 REGION 4         			   -511.596      186.392       -2.74473      [.006]
 REGION 6         			   410.755       271.255       1.51428       [.130]
 REGION 7         			   -113.106      279.488       -.404692      [.686]

 Standard Errors computed from heteroscedastic-consistent matrix
 (Robust-White)

        Mean of dep. var. = 6891.78               R-squared = .339203
   Std. dev. of dep. var. = 3390.54      Adjusted R-squared = .335233
 Sum of squared residuals = .203506E+11        LM het. test = 43.6254 [.000]
    Variance of residuals = .764200E+07       Durbin-Watson = 1.89016 [<.009]
 Std. error of regression = 2764.42
```

**Table 5. Regression results, total CO_2_ emissions: control variables regional dummy variables + child dummies for each child in different age groups**

```
                             NONLINEAR LEAST SQUARES
                             =======================

 Parameter  				Estimate        Error       t-statistic   P-value
 CONSTANT   				3815.59       364.338       10.4727       [.000]
 FIRST CHILD 1 TO 3 YERAS      	97.7537       213.309       .458273       [.647]
 SECOND CHILD 1 TO 3 YERAS      	126.958       356.455       .356167       [.722]
 THIRD CHILD 1 TO 3 YERAS      	-464.135      1162.52       -.399248      [.690]
 FIRST CHILD 4 TO 6 YERAS      	205.496       189.763       1.08291       [.279]
 SECOND CHILD 4 TO 6 YERAS      	1044.19       377.875       2.76331       [.006]
 FIRST CHILD 7 TO 12 YERAS     	441.094       191.448       2.30400       [.021]
 SECOND CHILD 7 TO 12 YERAS     	655.114       253.184       2.58750       [.010]
 THIRD CHILD 7 TO 12 YERAS     	1442.49       869.459       1.65907       [.097]
 FIRST CHILD 13 TO 17 YERAS    	633.085       199.176       3.17851       [.001]
 SECOND CHILD 13 TO 17 YERAS    	956.241       287.755       3.32311       [.001]
 THIRD CHILD 13 TO 17 YERAS    	667.132       806.370       .827327       [.408]
 FIRST CHILD 18 TO 19 YERAS    	-11.1176      242.132       -.045916      [.963]
 SECOND CHILD 18 TO 19 YERAS    	1157.54       1012.24       1.14354       [.253]
 TWO ADULTS WITHOUT CHILDREN     	-715.478      253.383       -2.82370      [.005]
 ONE ADULT WITHOUT CHILDREN     	-2269.27      274.654       -8.26229      [.000]
 ONE ADULT WITH CHILDREN   	-1854.31      210.652       -8.80271      [.000]
 AGE      				26.9278       5.30293       5.07790       [.000]
 DISP. INCOME         		7.28642       .680387       10.7092       [.000]
 DISP. INCOME SQUARED         	-.235083E-02  .273765E-03   -8.58704      [.000]
 REGION 1         			-815.679      172.248       -4.73548      [.000]
 REGION 2         			-419.302      179.064       -2.34164      [.019]
 REGION 3         			-84.6346      223.327       -.378971      [.705]
 REGION 4         			-517.478      186.822       -2.76989      [.006]
 REGION 6         			408.354       271.288       1.50524       [.132]
 REGION 7         			-129.116      280.252       -.460713      [.645]

 Standard Errors computed from heteroscedastic-consistent matrix
 (Robust-White)

        Mean of dep. var. = 6891.78               R-squared = .341395
   Std. dev. of dep. var. = 3390.54      Adjusted R-squared = .335191
 Sum of squared residuals = .202832E+11        LM het. test = 43.1651 [.000]
    Variance of residuals = .764248E+07       Durbin-Watson = 1.88575 [<.014]
 Std. error of regression = 2764.50
```

**Table 6. Regression results, CO_2_ emissions from food**

```
                             NONLINEAR LEAST SQUARES
                             =======================

Parameter  				  Estimate	    Error       t-statistic    P-value
 CONSTANT   				   1028.39       130.270       7.89433       [.000]
 NUMBER OF CHILDREN 0-6 YEARS       87.7251       48.8958       1.79412       [.073]
 NUMBER OF CHILDREN 7-12 YEARS      177.857       50.9366       3.49174       [.000]
 NUMBER OF CHILDREN 13-17 YEARS     365.017       50.9761       7.16055       [.000]
 NUMBER OF CHILDREN 8-19 YEARS      322.203       103.677       3.10776       [.002]
 TWO ADULTS WITHOUT CHILDREN     	   -303.971      92.8532       -3.27367      [.001]
 ONE ADULT WITHOUT CHILDREN     	   -924.352      97.5269       -9.47792      [.000]
 ONE ADULT WITH CHILDREN   	   -669.861      95.9633       -6.98039      [.000]
 AGE      				   10.5140       1.80850       5.81364       [.000]
 DISP. INCOME         		   2.29557       .262908       8.73146       [.000]
 DISP. INCOME SQUARED         	   -.659803E-03  .124966E-03   -5.27988      [.000]

 Standard Errors computed from heteroscedastic-consistent matrix
 (Robust-White)

        Mean of dep. var. = 2287.74               R-squared = .329430
   Std. dev. of dep. var. = 1398.22      Adjusted R-squared = .326928
 Sum of squared residuals = .352784E+10        LM het. test = 81.0648 [.000]
    Variance of residuals = .131587E+07       Durbin-Watson = 1.95809 [<.216]
 Std. error of regression = 1147.11
```

**Table 6. Regression results, CO_2_ emissions from food: control variables regional dummy variables**

```
                             NONLINEAR LEAST SQUARES
                             =======================

 Parameter  				Estimate        Error       t-statistic   P-value
 CONSTANT   				1045.34       137.643       7.59459       [.000]
 NUMBER OF CHILDREN 0-6 YEARS    86.5139       48.9674       1.76677       [.077]
 NUMBER OF CHILDREN 7-12 YEARS   174.181       50.7747       3.43047       [.001]
 NUMBER OF CHILDREN 13-17 YEARS  366.708       51.1669       7.16691       [.000]
 NUMBER OF CHILDREN 8-19 YEARS   314.541       102.759       3.06097       [.002]
 TWO ADULTS WITHOUT CHILDREN     	-306.971      92.9880       -3.30119      [.001]
 ONE ADULT WITHOUT CHILDREN     	-928.454      97.3730       -9.53503      [.000]
 ONE ADULT WITH CHILDREN   	-673.727      95.6112       -7.04652      [.000]
 AGE      				10.4986       1.80840       5.80548       [.000]
 DISP. INCOME         		2.32985       .262147       8.88756       [.000]
 DISP. INCOME SQUARED         	-.672975E-03  .126197E-03   -5.33274      [.000]
 REGION 1        			-56.2540      74.3099       -.757019      [.449]
 REGION 2         			-76.6908      74.9708       -1.02294      [.306]
 REGION 3         			137.175       96.8639       1.41616       [.157]
 REGION 4         			-83.2911      73.9933       -1.12566      [.260]
 REGION 6         			91.8691       103.668       .886186       [.376]
 REGION 7         			31.9385       135.744       .235284       [.814]

 Standard Errors computed from heteroscedastic-consistent matrix
 (Robust-White)

        Mean of dep. var. = 2287.74               R-squared = .332049
   Std. dev. of dep. var. = 1398.22      Adjusted R-squared = .328054
 Sum of squared residuals = .351405E+10        LM het. test = 84.7772 [.000]
    Variance of residuals = .131366E+07       Durbin-Watson = 1.95733 [<.268]
 Std. error of regression = 1146.15
```

**Table 6. Regression results, CO_2_ emissions from food: control variables regional dummy variables + child dummies for each child in different age groups**

```
                             NONLINEAR LEAST SQUARES
                             =======================

Parameter  				Estimate        Error       t-statistic   P-value
 CONSTANT   				1094.81       141.549       7.73452       [.000]
 FIRST CHILD 1 TO 3 YERAS      	51.3936       81.7246       .628863       [.529]
 SECOND CHILD 1 TO 3 YERAS      	-21.3343      141.086       -.151215      [.880]
 THIRD CHILD 1 TO 3 YERAS      	-644.795      884.230       -.729216      [.466]
 FIRST CHILD 4 TO 6 YERAS      	87.7661       77.4375       1.13338       [.257]
 SECOND CHILD 4 TO 6 YERAS      	617.142       151.625       4.07018       [.000]
 FIRST CHILD 7 TO 12 YERAS     	56.8562       74.4897       .763277       [.445]
 SECOND CHILD 7 TO 12 YERAS     	353.341       117.203       3.01477       [.003]
 FIRST CHILD 7 TO 12 YERAS     	856.803       441.360       1.94128       [.052]
 THIRD CHILD 13 TO 17 YERAS    	424.565       89.0441       4.76803       [.000]
 SECOND CHILD 13 TO 17 YERAS    	741.472       114.831       6.45708       [.000]
 THIRD CHILD 13 TO 17 YERAS    	774.332       376.131       2.05868       [.040]
 FIRST CHILD 18 TO 19 YERAS    	253.212       115.059       2.20071       [.028]
 SECOND CHILD 18 TO 19 YERAS    	987.866       444.827       2.22079       [.026]
 TWO ADULTS WITHOUT CHILDREN     	-336.846      100.113       -3.36467      [.001]
 ONE ADULT WITHOUT CHILDREN     	-960.501      103.860       -9.24808      [.000]
 ONE ADULT WITH CHILDREN   	-685.145      95.5991       -7.16686      [.000]
 AGE      				10.1341       1.80776       5.60589       [.000]
 DISP. INCOME         		2.33205       .259196       8.99724       [.000]
 DISP. INCOME SQUARED         	-.677513E-03  .122589E-03   -5.52669      [.000]
 REGION 1         			-54.5439      74.6685       -.730481      [.465]
 REGION 2         			-80.9744      74.9588       -1.08025      [.280]
 REGION 3         			129.717       96.2895       1.34716       [.178]
 REGION 4         			-85.0541      74.4477       -1.14247      [.253]
 REGION 6         			90.9196       103.800       .875916       [.381]
 REGION 7         			21.9079       136.956       .159963       [.873]

 Standard Errors computed from heteroscedastic-consistent matrix
 (Robust-White)

        Mean of dep. var. = 2287.74               R-squared = .337000
   Std. dev. of dep. var. = 1398.22      Adjusted R-squared = .330783
 Sum of squared residuals = .348801E+10        LM het. test = 85.0381 [.000]
    Variance of residuals = .130833E+07       Durbin-Watson = 1.94924 [<.291]
 Std. error of regression = 1143.82
```

**Table 7. Regression results, CO_2_ emissions from meat consumption**

```
     NONLINEAR LEAST SQUARES
                             =======================

 Parameter  				  Estimate        Error       t-statistic   P-value
 CONSTANT   				   295.613       86.6095       3.41318       [.001]
 NUMBER OF CHILDREN 0-6 YEARS       -16.9159      30.6803       -.551361      [.581]
 NUMBER OF CHILDREN 7-12 YEARS      35.4562       33.8581       1.04720       [.295]
 NUMBER OF CHILDREN 13-17 YEARS     128.366       33.4939       3.83252       [.000]
 NUMBER OF CHILDREN 8-19 YEARS      96.8431       68.2022       1.41994       [.156]
 TWO ADULTS WITHOUT CHILDREN     	   -121.903      62.1988       -1.95990      [.050]
 ONE ADULT WITHOUT CHILDREN     	   -333.468      65.0181       -5.12886      [.000]
 ONE ADULT WITH CHILDREN   	   -281.128      59.9944       -4.68591      [.000]
 AGE      			          3.57079       1.17207       3.04655       [.002]
 DISP. INCOME         		   1.35475       .190665       7.10538       [.000]
 DISP. INCOME SQUARED         	   -.357698E-03  .963475E-04   -3.71258      [.000]

 Standard Errors computed from heteroscedastic-consistent matrix
 (Robust-White)

        Mean of dep. var. = 898.478               R-squared = .173710
   Std. dev. of dep. var. = 835.827      Adjusted R-squared = .170628
 Sum of squared residuals = .155338E+10        LM het. test = 55.3333 [.000]
    Variance of residuals = 579405.           Durbin-Watson = 1.92056 [<.039]
 Std. error of regression = 761.187
```

**Table 7. Regression results, CO_2_ emissions from meat consumption: control variables regional dummy variables**

```
                             NONLINEAR LEAST SQUARES
                             =======================

 Parameter  				Estimate        Error       t-statistic   P-value
 CONSTANT   				320.343       89.7761       3.56824       [.000]
 NUMBER OF CHILDREN 0-6 YEARS    -16.4441      30.6663       -.536227      [.592]
 NUMBER OF CHILDREN 7-12 YEARS   32.2213       33.8091       .953036       [.341]
 NUMBER OF CHILDREN 13-17 YEARS  130.407       33.6498       3.87540       [.000]
 NUMBER OF CHILDREN 8-19 YEARS   90.4897       67.7513       1.33562       [.182]
 TWO ADULTS WITHOUT CHILDREN     	-122.397      62.0963       -1.97108      [.049]
 ONE ADULT WITHOUT CHILDREN     	-333.823      64.7422       -5.15618      [.000]
 ONE ADULT WITH CHILDREN   	-282.772      59.9722       -4.71505      [.000]
 AGE      				3.58698       1.17275       3.05860       [.002]
 DISP. INCOME         		1.38072       .190584       7.24467       [.000]
 DISP. INCOME SQUARED         	-.366498E-03  .972654E-04   -3.76802      [.000]
 REGION 1         			-67.0684      48.9494       -1.37016      [.171]
 REGION 2         			-70.6374      49.5489       -1.42561      [.154]
 REGION 3         			92.4068       63.7845       1.44873       [.147]
 REGION 4         			-69.6568      49.2424       -1.41457      [.157]
 REGION 6         			9.80824       73.8938       .132734       [.894]
 REGION 7         			17.5939       93.9577       .187254       [.851]

 Standard Errors computed from heteroscedastic-consistent matrix
 (Robust-White)

        Mean of dep. var. = 898.478               R-squared = .177672
   Std. dev. of dep. var. = 835.827      Adjusted R-squared = .172754
 Sum of squared residuals = .154594E+10        LM het. test = 58.8584 [.000]
    Variance of residuals = 577920.           Durbin-Watson = 1.91885 [<.053]
 Std. error of regression = 760.210
```

**Table 7. Regression results, CO_2_ emissions from meat consumption: Control regional and child dummies**

```
                             NONLINEAR LEAST SQUARES
                             =======================

 Parameter  				Estimate        Error       t-statistic   P-value
 CONSTANT   				360.269       93.6117       3.84855       [.000]
 FIRST CHILD 1 TO 3 YERAS      	-60.0771      51.4367       -1.16798      [.243]
 SECOND CHILD 1 TO 3 YERAS     	-127.218      79.6361       -1.59749      [.110]
 THIRD CHILD 1 TO 3 YERAS      	-579.273      367.973       -1.57422      [.115]
 FIRST CHILD 4 TO 6 YERAS      	-9.08268      49.6184       -.183051      [.855]
 SECOND CHILD 4 TO 6 YERAS      	190.957       102.656       1.86016       [.063]
 FIRST CHILD 7 TO 12 YERAS     	-31.4177      49.0453       -.640586      [.522]
 SECOND CHILD 7 TO 12 YERAS     	87.3282       79.3773       1.10017       [.271]
 THIRD CHILD 7 TO 12 YERAS     	55.6114       267.895       .207586       [.836]
 FIRST CHILD 13 TO 17 YERAS    	152.091       61.0687       2.49049       [.013]
 SECOND CHILD 13 TO 17 YERAS    	235.127       72.9151       3.22466       [.001]
 THIRD CHILD 13 TO 17 YERAS    	450.991       281.591       1.60158       [.109]
 FIRST CHILD 18 TO 19 YERAS   	41.3748       75.9056       .545083       [.586]
 SECOND CHILD 18 TO 19 YERAS    	441.453       314.946       1.40168       [.161]
 TWO ADULTS WITHOUT CHILDREN     	-150.239      66.9695       -2.24340      [.025]
 ONE ADULT WITHOUT CHILDREN     	-363.810      69.7157       -5.21848      [.000]
 ONE ADULT WITH CHILDREN   	-289.268      59.9768       -4.82300      [.000]
 AGE      				3.41372       1.17171       2.91345       [.004]
 DISP. INCOME         		1.37622       .189033       7.28036       [.000]
 DISP. INCOME SQUARED         	-.367785E-03  .947741E-04   -3.88064      [.000]
 REGION 1         			-65.2235      49.1015       -1.32834      [.184]
 REGION 2         			-73.9517      49.6872       -1.48834      [.137]
 REGION 3         			87.7191       63.5817       1.37963       [.168]
 REGION 4         			-72.7550      49.3406       -1.47455      [.140]
 REGION 6         			8.67767       73.9208       .117391       [.907]
 REGION 7         			14.0381       94.6473       .148320       [.882]

 Standard Errors computed from heteroscedastic-consistent matrix
 (Robust-White)

        Mean of dep. var. = 898.478               R-squared = .181389
   Std. dev. of dep. var. = 835.827      Adjusted R-squared = .173713
 Sum of squared residuals = .153895E+10        LM het. test = 59.0941 [.000]
    Variance of residuals = 577250.           Durbin-Watson = 1.91713 [<.083]
 Std. error of regression = 759.769
```

**Table 8. Regression results, CO_2_ emissions from transportation**

```
                             NONLINEAR LEAST SQUARES
                             =======================

 Parameter  				Estimate        Error       t-statistic   P-value
 CONSTANT   				  1378.02       271.889       5.06834     [.000]
 NUMBER OF CHILDREN 0-6 YEARS    88.7527       97.0800       .914222       [.361]
 NUMBER OF CHILDREN 7-12 YEARS   284.884       90.5386       3.14654       [.002]
 NUMBER OF CHILDREN 13-17 YEARS  159.581       104.275       1.53039       [.126]
 NUMBER OF CHILDREN 8-19 YEARS    3.82179       177.227       .021564      [.983]
 TWO ADULTS WITHOUT CHILDREN     	-396.324      201.471       -1.96715      [.049]
 ONE ADULT WITHOUT CHILDREN     	-1349.46      217.118       -6.21532      [.000]
 ONE ADULT WITH CHILDREN   	-1464.50      166.548       -8.79325      [.000]
 AGE      				23.7556       4.56354       5.20553       [.000]
 DISP. INCOME         		3.26923       .554393       5.89696       [.000]
 DISP. INCOME SQUARED         	-.109229E-02  .212708E-03   -5.13517      [.000]

 Standard Errors computed from heteroscedastic-consistent matrix
 (Robust-White)

        Mean of dep. var. = 3312.00               R-squared = .169707
   Std. dev. of dep. var. = 2591.94      Adjusted R-squared = .166610
 Sum of squared residuals = .150106E+11        LM het. test = 28.1033 [.000]
    Variance of residuals = .559886E+07       Durbin-Watson = 1.84438 [<.000]
 Std. error of regression = 2366.19
```

**Table 8. Regression results, CO_2_ emissions from transportation: control variables regional dummy variables**

```
                             NONLINEAR LEAST SQUARES
                             =======================

 Parameter  				Estimate        Error       t-statistic   P-value
 CONSTANT   				1689.41       285.619       5.91491       [.000]
 NUMBER OF CHILDREN 0-6 YEARS    83.2284       95.8409       .868402       [.385]
 NUMBER OF CHILDREN 7-12 YEARS   242.856       89.8751       2.70216       [.007]
 NUMBER OF CHILDREN 13-17 YEARS  132.875       103.037       1.28958       [.197]
 NUMBER OF CHILDREN 8-19 YEARS   -105.104      178.281       -.589539      [.555]
 TWO ADULTS WITHOUT CHILDREN     	-404.117      198.472       -2.03614      [.042]
 ONE ADULT WITHOUT CHILDREN     	-1259.88      212.596       -5.92615      [.000]
 ONE ADULT WITH CHILDREN   	-1373.70      164.501       -8.35068      [.000]
 AGE      				22.1231       4.52192       4.89241       [.000]
 DISP. INCOME         		3.85188       .559456       6.88506       [.000]
 DISP. INCOME SQUARED         	-.126905E-02  .213417E-03   -5.94634      [.000]
 REGION 1         			-1034.80      143.438       -7.21428      [.000]
 REGION 2         			-280.214      155.047       -1.80729      [.071]
 REGION 3         			-448.614      180.402       -2.48675      [.013]
 REGION 4         			-530.798      156.599       -3.38953      [.001]
 REGION 6         			500.078       238.682       2.09517       [.036]
 REGION 7         			-215.147      234.509       -.917437      [.359]

 Standard Errors computed from heteroscedastic-consistent matrix
 (Robust-White)

        Mean of dep. var. = 3312.00               R-squared = .196335
   Std. dev. of dep. var. = 2591.94      Adjusted R-squared = .191528
 Sum of squared residuals = .145291E+11        LM het. test = 36.4221 [.000]
    Variance of residuals = .543146E+07       Durbin-Watson = 1.85069 [<.000]
 Std. error of regression = 2330.55
```

**Table 8. Regression results, CO_2_ emissions from transportation: control variables regional dummy variables + child dummies for each child in different age groups**

```
                             NONLINEAR LEAST SQUARES
                             =======================

Parameter  				Estimate        Error       t-statistic   P-value
 CONSTANT   				1701.17       304.412       5.58838       [.000]
 FIRST CHILD 1 TO 3 YERAS      	9.44088       178.925       .052764       [.958]
 SECOND CHILD 1 TO 3 YERAS      	-29.3563      298.271       -.098422      [.922]
 THIRD CHILD 1 TO 3 YERAS      	417.042       307.763       1.35507       [.175]
 FIRST CHILD 4 TO 6 YERAS      	157.953       158.864       .994263       [.320]
 SECOND CHILD 4 TO 6 YERAS      	399.815       355.437       1.12486       [.261]
 FIRST CHILD 7 TO 12 YERAS    	365.520       162.582       2.24822       [.025]
 SECOND CHILD 7 TO 12 YERAS     	326.301       202.673       1.60999       [.107]
 THIRD CHILD 7 TO 12 YERAS     	963.778       610.344       1.57907       [.114]
 FIRST CHILD 13 TO 17 YERAS    	206.392       162.844       1.26742       [.205]
 SECOND CHILD 13 TO 17 YERAS    	228.299       239.985       .951303       [.341]
 THIRD CHILD 13 TO 17 YERAS    	91.2832       777.478       .117409       [.907]
 FIRST CHILD 18 TO 19 YERAS    	-182.776      194.062       -.941842      [.346]
 SECOND CHILD 18 TO 19 YERAS    	435.338       970.202       .448708       [.654]
 TWO ADULTS WITHOUT CHILDREN     	-380.437      213.019       -1.78593      [.074]
 ONE ADULT WITHOUT CHILDREN     	-1240.79      231.494       -5.35993      [.000]
 ONE ADULT WITH CHILDREN   	-1386.57      166.789       -8.31335      [.000]
 AGE      				21.6335       4.49084       4.81725       [.000]
 DISP. INCOME         		3.84109       .568516       6.75634       [.000]
 DISP. INCOME SQUARED         	-.126791E-02  .218180E-03   -5.81132      [.000]
 REGION 1         			-1036.34      143.596       -7.21700      [.000]
 REGION 2         			-298.088      155.094       -1.92199      [.055]
 REGION 3         			-465.033      178.625       -2.60340      [.009]
 REGION 4         			-538.538      156.385       -3.44366      [.001]
 REGION 6         			494.399       238.084       2.07657       [.038]
 REGION 7         			-220.804      235.777       -.936498      [.349]

 Standard Errors computed from heteroscedastic-consistent matrix
 (Robust-White)

        Mean of dep. var. = 3312.00               R-squared = .198258
   Std. dev. of dep. var. = 2591.94      Adjusted R-squared = .190740
 Sum of squared residuals = .144944E+11        LM het. test = 39.3068 [.000]
    Variance of residuals = .543675E+07       Durbin-Watson = 1.85000 [<.001]
 Std. error of regression = 2331.68
```

**Table 9. Regression results, CO_2_ emissions from gasoline**

```
                             NONLINEAR LEAST SQUARES
                             =======================

 Parameter  				  Estimate        Error       t-statistic   P-value
 CONSTANT   				   1170.88       275.877       4.24419       [.000]
 NUMBER OF CHILDREN 0-6 YEARS       127.991       97.7601       1.30923       [.190]
 NUMBER OF CHILDREN 7-12 YEARS      314.443       91.3803       3.44104       [.001]
 NUMBER OF CHILDREN 13-17 YEARS     177.974       104.700       1.69985       [.089]
 NUMBER OF CHILDREN 8-19 YEARS      6.58015       179.057       .036749       [.971]
 TWO ADULTS WITHOUT CHILDREN     	   -383.573      203.672       -1.88328      [.060]
 ONE ADULT WITHOUT CHILDREN     	   -1326.90      220.293       -6.02337      [.000]
 ONE ADULT WITH CHILDREN   	   -1488.90      170.025       -8.75692      [.000]
 AGE      				   26.0347       4.60582       5.65256       [.000]
 DISP. INCOME         		   3.08057       .558808       5.51276       [.000]
 DISP. INCOME SQUARED         	   -.105991E-02  .214798E-03   -4.93444      [.000]

 Standard Errors computed from heteroscedastic-consistent matrix
 (Robust-White)

        Mean of dep. var. = 3168.84               R-squared = .166251
   Std. dev. of dep. var. = 2608.75      Adjusted R-squared = .163141
 Sum of squared residuals = .152692E+11        LM het. test = 26.3155 [.000]
    Variance of residuals = .569533E+07       Durbin-Watson = 1.84580 [<.000]
 Std. error of regression = 2386.49
```

**Table 9. Regression results, CO_2_ emissions from gasoline: control variables regional dummy variables**

```
                             NONLINEAR LEAST SQUARES
                             =======================

Parameter  				  Estimate        Error       t-statistic   P-value
 CONSTANT   				   1522.99       287.082       5.30507       [.000]
 NUMBER OF CHILDREN 0-6 YEARS       123.063       96.1307       1.28016       [.200]
 NUMBER OF CHILDREN 7-12 YEARS      263.560       90.2901       2.91904       [.004]
 NUMBER OF CHILDREN 13-17 YEARS     145.701       103.159       1.41239       [.158]
 NUMBER OF CHILDREN 8-19 YEARS     -124.991      179.790       -.695205       [.487]
 TWO ADULTS WITHOUT CHILDREN     	   -389.492      199.588       -1.95148      [.051]
 ONE ADULT WITHOUT CHILDREN     	   -1214.60      214.137       -5.67205      [.000]
 ONE ADULT WITH CHILDREN   	   -1375.78      166.206       -8.27757      [.000]
 AGE      				   24.1289       4.53555       5.31995       [.000]
 DISP. INCOME         		   3.78021       .558841       6.76437       [.000]
 DISP. INCOME SQUARED         	   -.126908E-02  .210984E-03   -6.01508      [.000]
 REGION 1         			   -1246.91      143.049       -8.71666      [.000]
 REGION 2         			   -293.563      154.782       -1.89662      [.058]
 REGION 3         			   -509.802      180.899       -2.81816      [.005]
 REGION 4         			   -583.636      155.825       -3.74547      [.000]
 REGION 6         			   535.656       238.995       2.24129       [.025]
 REGION 7         			   -260.356      236.680       -1.10003      [.271]

 Standard Errors computed from heteroscedastic-consistent matrix
 (Robust-White)

        Mean of dep. var. = 3168.84               R-squared = .203193
   Std. dev. of dep. var. = 2608.75      Adjusted R-squared = .198427
 Sum of squared residuals = .145926E+11        LM het. test = 34.2341 [.000]
    Variance of residuals = .545519E+07       Durbin-Watson = 1.85268 [<.000]
 Std. error of regression = 2335.63
```

**Table 9. Regression results, CO_2_ emissions from gasoline: control variables regional dummy variables + child dummies for each child in different age groups**

```
                             NONLINEAR LEAST SQUARES
                             =======================

Parameter  				Estimate        Error       t-statistic   P-value
 CONSTANT   				1527.77       306.003       4.99266       [.000]
 FIRST CHILD 1 TO 3 YERAS      	67.8356       179.276       .378385       [.705]
 SECOND CHILD 1 TO 3 YERAS      	51.8087       297.556       .174114       [.862]
 THIRD CHILD 1 TO 3 YERAS      	525.107       314.067       1.67196       [.095]
 FIRST CHILD 4 TO 6 YERAS      	180.589       158.688       1.13802       [.255]
 SECOND CHILD 4 TO 6 YERAS      	477.063       357.720       1.33362       [.182]
 FIRST CHILD 7 TO 12 YERAS     	389.076       163.053       2.38619       [.017]
 SECOND CHILD 7 TO 12 YERAS     	375.146       202.378       1.85368       [.064]
 THIRD CHILD 7 TO 12 YERAS     	1028.54       625.096       1.64541       [.100]
 FIRST CHILD 13 TO 17 YERAS    	202.983       163.781       1.23936       [.215]
 SECOND CHILD 13 TO 17 YERAS    	262.645       239.766       1.09542       [.273]
 THIRD CHILD 13 TO 17 YERAS    	176.696       786.396       .224692       [.822]
 FIRST CHILD 18 TO 19 YERAS    	-190.488      195.253       -.975592      [.329]
 SECOND CHILD 18 TO 19 YERAS    	332.478       996.412       .333675       [.739]
 TWO ADULTS WITHOUT CHILDREN     	-365.161      214.060       -1.70588      [.088]
 ONE ADULT WITHOUT CHILDREN     	-1193.51      233.118       -5.11977      [.000]
 ONE ADULT WITH CHILDREN   	-1386.10      168.631       -8.21971      [.000]
 AGE      				23.7306       4.50263       5.27039       [.000]
 DISP. INCOME         		3.77444       .567964       6.64556       [.000]
 DISP. INCOME SQUARED         	-.126951E-02  .215209E-03   -5.89893      [.000]
 REGION 1         			-1248.97      143.178       -8.72322      [.000]
 REGION 2         			-310.702      154.736       -2.00794      [.045]
 REGION 3         			-525.645      179.051       -2.93573      [.003]
 REGION 4         			-591.486      155.573       -3.80199      [.000]
 REGION 6         			530.274       238.368       2.22460       [.026]
 REGION 7         			-266.442      237.843       -1.12024      [.263]

 Standard Errors computed from heteroscedastic-consistent matrix
 (Robust-White)

        Mean of dep. var. = 3168.84               R-squared = .204860
   Std. dev. of dep. var. = 2608.75      Adjusted R-squared = .197403
 Sum of squared residuals = .145621E+11        LM het. test = 36.7845 [.000]
    Variance of residuals = .546215E+07       Durbin-Watson = 1.85231 [<.001]
 Std. error of regression = 2337.12
```

**Table 10. Regression results, CO_2_ emissions from electricity and heating**

```
                             NONLINEAR LEAST SQUARES
                             =======================

 Parameter  				  Estimate        Error       t-statistic   P-value
 CONSTANT   				  797.019       94.7247       8.41406       [.000]
 NUMBER OF CHILDREN 0-6 YEARS      65.8858       41.1184       1.60234       [.109]
 NUMBER OF CHILDREN 7-12 YEARS     -1.18994      33.7696       -.035237      [.972]
 NUMBER OF CHILDREN 13-17 YEARS    20.4811       33.3415       .614281       [.539]
 NUMBER OF CHILDREN 8-19 YEARS     -96.4277      48.6637       -1.98151      [.048]
 TWO ADULTS WITHOUT CHILDREN     	  17.7050       65.7384       .269325       [.788]
 ONE ADULT WITHOUT CHILDREN     	  -42.4245      66.6305       -.636712      [.524]
 ONE ADULT WITH CHILDREN   	  217.947       52.7433       4.13221       [.000]
 AGE      				  .560582       1.22008       .459462       [.646]
 DISP. INCOME         		  .148288       .171999       .862147       [.389]
 DISP. INCOME SQUARED         	  -.702363E-04  .705000E-04   -.996260      [.319]

 Standard Errors computed from heteroscedastic-consistent matrix
 (Robust-White)

        Mean of dep. var. = 905.403               R-squared = .011715
   Std. dev. of dep. var. = 785.463      Adjusted R-squared = .802848E-02
 Sum of squared residuals = .164077E+10        LM het. test = 7.82759 [.005]
    Variance of residuals = 611998.           Durbin-Watson = 2.00413 [<.658]
 Std. error of regression = 782.303
```

**Table 10. Regression results, CO_2_ emissions from electricity and heating: control variables regional dummy variables + child dummies for each child in different age groups**

```
                             NONLINEAR LEAST SQUARES
                             =======================

 Parameter  				Estimate        Error       t-statistic   P-value
 CONSTANT   				751.857       104.787       7.17510       [.000]
 NUMBER OF CHILDREN 0-6 YEARS    67.9873       40.5433       1.67691       [.094]
 NUMBER OF CHILDREN 7-12 YEARS   5.56452       33.8642       .164318       [.869]
 NUMBER OF CHILDREN 13-17 YEARS  29.8390       33.0953       .901606       [.367]
 NUMBER OF CHILDREN 8-19 YEARS  -76.2127      49.0268       -1.55451       [.120]
 TWO ADULTS WITHOUT CHILDREN     	19.6047       65.3089       .300185       [.764]
 ONE ADULT WITHOUT CHILDREN     	-65.2117      64.8336       -1.00583      [.314]
 ONE ADULT WITH CHILDREN   	192.537       52.1709       3.69050       [.000]
 AGE      				.965338       1.22815       .786012       [.432]
 DISP. INCOME         		.034419       .169956       .202518       [.840]
 DISP. INCOME SQUARED         	-.372518E-04  .687616E-04   -.541753      [.588]
 REGION 1         			176.171       50.3142       3.50143       [.000]
 REGION 2         			-22.4745      47.9452       -.468754      [.639]
 REGION 3         			226.400       70.5036       3.21118       [.001]
 REGION 4         			47.1348       50.2389       .938213       [.348]
 REGION 6         			-105.033      53.3322       -1.96941      [.049]
 REGION 7         			50.9531       67.7871       .751663       [.452]

 Standard Errors computed from heteroscedastic-consistent matrix
 (Robust-White)

        Mean of dep. var. = 905.403               R-squared = .027179
   Std. dev. of dep. var. = 785.463      Adjusted R-squared = .021360
 Sum of squared residuals = .161509E+10        LM het. test = 13.9027 [.000]
    Variance of residuals = 603774.           Durbin-Watson = 1.99922 [<.680]
 Std. error of regression = 777.029
```

**Table 10. Regression results, CO_2_ emissions from electricity and heating: control variables regional dummy variables + child dummies for each child in different age groups**

```
                             NONLINEAR LEAST SQUARES
                             =======================

 Parameter  				Estimate        Error       t-statistic   P-value
 CONSTANT   				712.499       109.264       6.52090       [.000]
 FIRST CHILD 1 TO 3 YERAS      	107.880       67.4203       1.60011       [.110]
 SECOND CHILD 1 TO 3 YERAS      	305.780       184.973       1.65311       [.098]
 THIRD CHILD 1 TO 3 YERAS      	-55.5740      272.494       -.203946      [.838]
 FIRST CHILD 4 TO 6 YERAS      	18.4590       56.8366       .324773       [.745]
 SECOND CHILD 4 TO 6 YERAS      	64.1235       102.502       .625581       [.532]
 FIRST CHILD 7 TO 12 YERAS     	39.2912       56.9878       .689467       [.491]
 SECOND CHILD 7 TO 12 YERAS     	31.1026       72.1978       .430797       [.667]
 THIRD CHILD 7 TO 12 YERAS     	-92.1588      269.221       -.342316      [.732]
 FIRST CHILD 13 TO 17 YERAS    	55.8053       58.2626       .957822       [.338]
 SECOND CHILD 13 TO 17 YERAS    	57.3695       76.6287       .748668       [.454]
 THIRD CHILD 13 TO 17 YERAS    	-18.9745      152.633       -.124314      [.901]
 FIRST CHILD 18 TO 19 YERAS    	-67.4299      58.6557       -1.14959      [.250]
 SECOND CHILD 18 TO 19 YERAS    	-268.606      131.067       -2.04937      [.040]
 TWO ADULTS WITHOUT CHILDREN     	40.6957       74.1493       .548834       [.583]
 ONE ADULT WITHOUT CHILDREN     	-39.3901      73.6959       -.534495      [.593]
 ONE ADULT WITH CHILDREN   	199.834       52.1679       3.83058       [.000]
 AGE      				1.08132       1.20930       .894174       [.371]
 DISP. INCOME         		.059913       .169140       .354219       [.723]
 DISP. INCOME SQUARED         	-.464137E-04  .679451E-04   -.683105      [.495]
 REGION 1         			178.748       50.5278       3.53763       [.000]
 REGION 2         			-18.6744      48.2299       -.387195      [.699]
 REGION 3         			231.909       72.0988       3.21655       [.001]
 REGION 4         			52.6649       50.8155       1.03640       [.300]
 REGION 6         			-99.2427      53.6768       -1.84889      [.064]
 REGION 7         			52.8475       68.6522       .769785       [.441]

 Standard Errors computed from heteroscedastic-consistent matrix
 (Robust-White)

        Mean of dep. var. = 905.403               R-squared = .029661
   Std. dev. of dep. var. = 785.463      Adjusted R-squared = .020561
 Sum of squared residuals = .161097E+10        LM het. test = 22.5670 [.000]
    Variance of residuals = 604266.           Durbin-Watson = 1.99682 [<.753]
 Std. error of regression = 777.346
```
